# Superintendent Barker

**Published:** 1892-08

**Authors:** 


### SUPERINTENDENT BARKER.

### SENTIMENT VS. DUTY.

The County Commissioners of this (Travis) county are after Superintendent Barker of the Southwest Texas Insane Asylum with a sharp stick, so to speak. They have addressed a communication to Governor Hogg in which complaint is made that the county jails are full of pauper insane, while there is room in the Southwest Texas Asylum; and in dealing with the subject they fairly knock the bark off; they give Dr. Barker emphatically to understand that he is "barking up the wrong tree" when he tries to shut the eyes of these commissioners with any sentiment.

Their complaint is general and specific; they mention Henry Purnell particularly, and represent to the Governor that this man is in jail—having been adjudged insane—and that application to Dr. Barker for his admission to the Southwest Texas Lunatic Asylum is refused, on the ground that Purnell killed "a friend of his," and that it would be unpleasant to look at him.

Dr. Reeves was not only a friend of Dr. Barker's, but he was a friend of the county commissioners and of the county jailor, and of everybody who knew him, and his untimely death was universally deplored. But, it was the act of a madman, and such sentiment as Dr. Barker gives as an excuse for refusing him admission into the State Asylum is puerile, and unbecoming a man of ordinary common sense. The law gives precedence to indigent insane, and they are to be admitted in preference to the wealthy class, who, it is supposed, can find asylum elsewhere; and the Commissioners in their memorial to the Governor set forth that Dr. Barker has an abundance of room, and ask for a mandamus, as it were, to compel him to subordinate his personal feelings to his duty and to admit Purnell. Poor Purnell! We wonder if the sight of him is more "unpleasant" to the fastidious doctor, than it is to Sheriff White? Or more unpleasant to either of them, than are the walls of a filthy prison to him?

Since this refusal to admit Purnell, the fire at the Austin Asylum occurred, and seventy-five women patients were unhoused. We learn that Dr. Barker refused to receive them, or any of them, temporarily, pending the repairing of their wards, alleging that "the law" [the devil quoting Scripture] gives his space to the pauper insane who are now in jail! and that there are enough insane in jail to more than fill his Asylum! The capacity of the Asylum is said to be two hundred, and the number of patients now on hand one hundred. While he urges that he is reserving his space for the indigent insane, the Doctor allows them to still languish in jail.

That this is a mere pretext, and to show that Dr. Barker could easily, and without violating the law, have relieved Superintendent White (Austin) of a part of his unhoused patients, had the inclination been present, and also as illustrating the difference between man in such positions; that there are Superintendents and Superintendents, the fact is recalled that when application was made to him to receive a portion of these people and he was assured that they could not, without uncomfortable and perhaps dangerous crowding, be cared for in the main Asylum at Austin, he *came up to Austin to see for himself* if the truth had been told him; to satisfy himself that no other arrangement could be made before calling on him! We will let our readers give it a name; but we must assume that he came to the conclusion that the facts had not been fairly stated and that Superintendent White *could* take care of these patients if he would, as he refused, and, on the pretext given; Superintendent White having no other alternative *did* make provision by crowding them all into other wards, greatly to the discomfort of all. On the other hand, in further illustration of the fact that there are Superintendents, like men, "of many minds," Supt. John Preston, in charge of the North Texas Hospital for the Insane, at Terrell, did not wait to be asked, but on hearing of the fire telegraphed to Supt. White to know if he could be of any service, and placed ten beds—all he had—at Dr. White's disposal!

We presume Dr. Barker has some reason for his remarkable conduct other than that their presence would be "unpleasant" to him, or that he is saving his room for those in jail; if he has, our columns are open to him, and he is invited to use them in explaining what, without explanation, looks like a good sized outrage, malfeasance in office, and total unfitness for his high trust. Unless he can give a reason, and a very good one, he should be removed, and thus be made to realize that in the construction of this diverticulum for the excess of insane over accommodation, and its maintenance at the expense of the State, *his feelings* did not enter into the consideration, and hence no provision was made for "sentiment."

